# Effects of Different Chromium Compounds on Hematology and Inflammatory Cytokines in Rats Fed High-Fat Diet

**DOI:** 10.3389/fimmu.2021.614000

**Published:** 2021-02-26

**Authors:** Wojciech Dworzański, Iwona Sembratowicz, Ewelina Cholewińska, Krzysztof Tutaj, Bartosz Fotschki, Jerzy Juśkiewicz, Katarzyna Ognik

**Affiliations:** ^1^ Chair and Department of Human Anatomy, Medical University of Lublin, Lublin, Poland; ^2^ Department of Biochemistry and Toxicology, Faculty of Animal Sciences and Bioeconomy, University of Life Sciences in Lublin, Lublin, Poland; ^3^ Division of Food Science, Institute of Animal Reproduction and Food Research of the Polish Academy of Sciences, Olsztyn, Poland

**Keywords:** immunity, hematology, chromium picolinate, chromium(III)-methionine, chromium nanoparticles, cytokine, rat

## Abstract

The aim of the study was to determine how a high-fat diet supplemented with various forms of chromium affects hematological and immune parameters of the blood of rats. The rats received a standard diet or a high-fat diet supplemented with chromium at 0.3 mg/kg body weight (BW) in the form of chromium(III) picolinate, chromium(III)-methionine or nano-sized chromium. Selected hematological parameters were determined in the blood of the rats, including total white blood cell (WBC) count, leukogram, red blood cell (RBC) count, hemoglobin level (HGB), hematocrit (HCT), platelet count (PLT) and platelet percentage (PCT), as well as immune parameters: levels of immunoglobulins A and E (IgA and IgE), interleukin-6 (IL-6), interleukin-2 (IL-2), and tumor necrosis factor α (TNF-α); activity of ceruloplasmin (Cp); and levels of caspase 3 and 8 (Casp3 and Casp8). Feeding rats a high-fat diet increased blood markers of induction of inflammation, ie pro-inflammatory cytokines IL-6 and TNF-α, and also significantly increased IgE. The diet had no effect on the blood count, except for an increase in the number of neutrophils. The chromium compounds tested, particularly Cr-Met and Cr-NPs, stimulated the immune system of the rats, as indicated by increased concentrations of IgA, IgE, IL-2, IL-6, TNF-α, and Cp. Given the increase in inflammatory mediators induced by chromium, it should not be used to mitigate the effects of a high-fat diet. Moreover, chromium picolinate and chromium nanoparticles were shown to increase the content of caspase 3 and 8 in the blood of rats, which indicates a pro-apoptotic effect. The effects of the use of chromium nanoparticles include reductions in the WBC count and in the thrombocyte count (leuko- and thrombopenia). Taking account these data the use of chromium as dietary supplement should be reconsidered.

## Introduction

Chromium(III) is a microelement with a well-documented beneficial effect on carbohydrate and lipid metabolism, mainly through improvement of insulin signaling (1). Together with niacin, glutamic acid, glycine and cysteine, trivalent chromium is a component of glucose tolerance factor, which is responsible for insulin binding to the receptor, thereby enabling proper utilization of glucose by tissues (2). Furthermore, it increases the number of insulin receptors and insulin receptor activity through phosphorylation (3, 4). In addition, CrIII takes part in protein biosynthesis and transformations of nucleic acids (5, 6). A deficiency of this metal in the body results in reduced tissue sensitivity to insulin, disturbances in glucose tolerance, growth inhibition, and a deterioration in blood lipid parameters (7). Supplementation with chromium compounds can be an effective element of prevention and supportive therapy of diabetes and other illnesses, e.g. cardiovascular disease (8). There are often dietary factors underlying these diseases, resulting in overweight and obesity. Chromium can help to reduce body weight and improve the blood lipid profile, and for this reason it is a common ingredient in supplements used in weight loss treatment (9, 10). Both diabetes and obesity are accompanied by oxidative stress and chronic inflammation (11–13).

Chromium can have a beneficial effect on redox status by increasing the level of reduced glutathione (14), one of the most important endogenous antioxidants, as well as on the activity of the antioxidant enzymes superoxide dismutase and catalase (14, 15). In diabetes patients and animals with experimentally induced diabetes, chromium supplementation has been shown to reduce the level of oxidative stress markers and pro-inflammatory cytokines (15–17). According to Cheng et al. (18), the antioxidant activity of Cr depends on health status; in people with glucose intolerance it has an antioxidant effect, but in healthy individuals it can act as a pro-oxidant. The antioxidant and anti-inflammatory effects of chromium have been demonstrated by Chen et al. (19) in mice on a high-fat diet. The use of chromium was shown to minimize the negative effects of this type of diet and inhibit the progression of diet-induced fatty liver. Benefits in the form of reduced inflammation and oxidative stress in rats on a high-fat diet have also been shown in studies using additives of various chromium compounds, e.g. picolinate, nicotinate and histidinate (20, 21).

Many experiments have shown that chromium can act as an immune modulator (stimulatory or suppressive) through its effect on various immune system components, e.g. B cells, T cells, and macrophages, as well as on cytokine production (22–25). A deficiency of this metal in the body results in impaired humoral immunity (26). The ability of chromium to reduce the cortisol level may be partly responsible for its immunostimulatory effect. This hormone exerts a suppressive effect on antibody production and the functions of lymphocytes, as well as other leukocyte populations (25).

Long-term exposure or high doses of chromium, however, can have adverse cytotoxic and genotoxic effects, which may cause disturbances in immune mechanisms (22). There are reports indicating induction of oxidative stress by chromium compounds (27, 28). It is also worth bearing in mind that Cr can limit absorption of zinc or iron from the digestive tract (due to the existence of a common protein carrier), leading to a deficiency of these minerals and to anemia (26).

The most common form of chromium in dietary supplements for people and animals is picolinate, but it is also used in the form of selenium yeast or complexes with various organic ligands, such as nicotinate, certain amino acids, or organic acids, or as a biomimetic (29). Inorganic forms of this metal used as additives include chromium chloride, and recently chromium in nanoparticle form is gaining significant popularity (30–32). The various forms differ in terms of digestibility and biological activity, but also in their potential toxicity (20, 33).

In the present study the hypothesis was put forth that the use of a high-fat diet induces inflammation and can lead to disturbances in blood count. It was postulated that the use of the recommended level of chromium would counteract the negative effects of the experimental diet without adversely affecting the hematological parameters of the blood. To verify this hypothesis, an experiment was conducted to compare the effects of three chemical forms of chromium, i.e. chromium picolinate, a chromium methionine complex, and chromium nanoparticles.

## Material and Methods

### Forms of Chromium Used in the Experiment

Chromium picolinate (Cr-Pic; purity > 980 g/kg) was purchased from Sigma-Aldrich Sp. z o. o. (Poznan, Poland). Chromium methionine complex (Cr-Met) was purchased from Innobio Co., Ltd. (Siheung, South Korea). Chromium nanopowder (Cr-NPs) with 99.9% purity, size 60 to 80 nm, spherical shape, specific surface area 6 to 8 m^2^/g, bulk density 0.15 g/cm^3^, and true density 8.9 g/cm^3^ was purchased from SkySpring Nanomaterials (Houston, TX, USA).

### Animals and Diets

The experiment was conducted on 56 male Wistar outbred rats (Rattus norvegicus, Cmdb : WI). The animals were used in compliance with the European Guidelines for the Care and Use of Laboratory Animals (34). The experimental protocol was approved by the Local Animal Care and Use Committee (Approval No. 04/2019; Olsztyn, Poland). All efforts were made to minimize the suffering of the experimental animals. At the start of the experiment, rats aged 5 weeks and weighing 131 ± 4.33 g were randomly assigned to one of eight groups of seven rats each. The animals were kept individually in metabolic cages under a stable temperature (21–22°C), a 12:12 h light/dark cycle, and a ventilation rate of 20 air changes per hour. For 8 weeks, the rats had free access to tap water and semi-purified diets, which were prepared and then stored at 4°C in hermetic containers until the end of the experiment (**Table 1**). The diets were modifications of a casein diet for laboratory rodents recommended by the American Institute of Nutrition (35). Two types of diet were used: a standard diet (diet S) containing 8% rapeseed oil and 5% cellulose as sources of fat and dietary fiber, and a high-fat, low-fiber diet (diet F), which was a modification of diet S with 17% lard added in place of corn starch and cellulose content reduced to 3%. All diets were balanced for content of dietary protein from a casein preparation (20% of diet; Lacpol Co., Murowana Goslina, Poland) and dl-methionine (0.3% of diet), whereas the F diet had 23% higher caloric density than the S diet due to the enhanced fat content (25% vs 8% of diet). Various chromium sources prepared at laboratory scale were added to the S and F diets at a single dosage, for a two-factorial experimental design (see description of statistical analyses). The level of chromium administered to each rat was 0.3 mg/kg BW, selected according to the EFSA NDA Panel (36). The sources of dietary Cr were chromium(III) picolinate (Cr-Pic), chromium(III) methionine (Cr-Met), and nano-sized chromium (Cr-NPs). Although chromium in the form of nanoparticles is undoubtedly absorbed in the body better than other sources of this element, the recommended dose of CrNPs was also used in the experiment. It was dictated by the fact that the conducted studies were of a pilot nature and were to demonstrate the effect of all three used forms of chromium administered in identical recommended doses in order to reliably compare their impact on selected hematological and immunological parameters. For the safety of the operator preparing the experimental diets, especially the Cr-NPs preparation, all Cr sources (to maintain comparable conditions) were added to the diet as an emulsion together with dietary rapeseed oil rather than in a mineral mixture.

**Table 1:** Composition of diets fed to rats, %.

Ingredient/Group	S	F
Casein^1^	20.0	20.0
DL-methionine	0.3	0.3
Cellulose^2^	5.0	3.00
Sucrose	10.0	10.0
Rapeseed oil	8.0	8.0
Lard	–	17.0
Vitamin mixture^3^	1.0	1.0
Mineral mixture^4^	3.5	3.5
Choline chloride	0.2	0.2
Cholesterol	0.3	0.3
Corn starch^5^	51.7	36.7

^1^Casein preparation (LACPOL Co., Murowana Goslina, Poland), crude protein 89.7%, crude fat 0.3%, ash 2.0%, and water 8.0%.

^2^ α-cellulose (SIGMA, Poznan, Poland), main source of dietary fiber.

^3^AIN-93G-VM^36^, g/kg mix: 3.0 nicotinic acid, 1.6 Ca pantothenate, 0.7 pyridoxine-HCl, 0.6 thiamin-HCl, 0.6 riboflavin, 0.2 folic acid, 0.02 biotin, 2.5 vitamin B_12_ (cyanocobalamin, 0.1% in mannitol), 15.0 vitamin E (all-rac-α-tocopheryl acetate, 500 IU/g), 0.8 vitamin A (all-trans-retinyl palmitate, 500 000 IU/g), 0.25 vitamin D_3_ (cholecalciferol, 400 000 IU/g), 0.075 vitamin K_1_ (phylloquinone), 974.655 powdered sucrose.

^4^ Mineral mix, g/kg mix: 357 calcium carbonate anhydrous CaCO_3_, 196 dipotassium phosphate K_2_HPO_4_, 70.78 potassium citrate C_6_H_5_K_3_O_7_, 74 sodium chloride NaCl, 46.6 potassium sulfate K_2_SO_4_, 24 magnesium oxide MgO, 18 microelement mixture^5^, starch to 1 kg = 213.62.

^5^ Corn starch preparation: crude protein 0.6%, crude fat 0.9%, ash 0.2%, total dietary fiber 0%, water 8.8%. Microelements mixture, g/kg mix: 31 iron(III) citrate (16.7% Fe), 4.5 zinc carbonate ZnCO_3_ (56% Zn), 23.4 manganese (II) carbonate MnCO_3_ (44.4% Mn), copper carbonate CuCO_3_ (55.5% Cu), 0.04 potassium iodide KI, citric acid C_6_H_8_O_7_ to 100 g.

The experimental sources of dietary Cr, chromium(III) picolinate (Cr-Pic), chromium(III) methionine (Cr-Met), and nano-sized chromium (Cr-NPs), were added to the diet as an emulsion together with dietary rapeseed oil rather than in a mineral mixture (MX).

S—standard diet; F - high-fat, low-fiber diet.

### Sample Collection and Analyses

At the end of the experiment, the rats were fasted for 12 h and anesthetized *i.p*. with ketamine and xylazine (K, 100/kg BW; X, 10 mg/kg BW) according to recommendations for anesthesia and euthanasia of experimental animals. Following laparotomy, blood samples were drawn from the caudal vena cava into heparinized tubes, and finally the rats were euthanized by cervical dislocation. The blood plasma was prepared by solidification and low-speed centrifugation (350*g*, 10 min, 4°C). Plasma samples were kept frozen at −70°C until assay.

### *Ex Vivo* Analysis

The following hematological parameters were determined in whole heparinized blood using the ABACUS Jr VET Analyzer (DIATRON MI PLC, Budapest, Hungary): total white blood cell (WBC) count, lymphocyte (LYM) count and percentage, medium-sized cell (MID) count and percentage, neutrophils (NEU) count and percentage, red blood cell count (RBC), hemoglobin (HGB), hematocrit (HCT), mean corpuscular volume (MCV), mean corpuscular hemoglobin (MCH), mean corpuscular hemoglobin concentration (MCHC), red cell distribution width (RDWc), platelet count (PLT), platelet percentage (PCT), mean platelet volume (MPV), and platelet distribution width (PDWc).

In addition, selected immune parameters were determined in the blood plasma: levels of immunoglobulins A and E (IgA and IgE), interleukin-6 (IL-6), interleukin-2 (IL-2), and tumor necrosis factor α (TNF-α); activity of ceruloplasmin (Cp); and levels of caspase 3 and 8 (Casp3 and Casp8). Immune parameters were determined using commercial measurement enzyme-linked immunosorbent assay (ELISA) kit (MyBioSource Inc., San Diego, USA). Absorbances were measured at 450 nm *via* ELISA reader.

### Statistical Analysis

The results are expressed as means and pooled SEM. Two-way analysis of variance (ANOVA) was used to determine the effect of the Cr source (Cr: none, Cr-Pic, Cr-Met, Cr-NPs) and the diet type (D: standard or high-fat low-fiber diets) and the interaction between these two factors (Cr × D). If the analysis revealed a significant interaction (*P* ≤ 0.05), the differences between treatment groups were then determined by Duncan’s *post hoc* test at *P* ≤ 0.05. The data were checked for normality prior to the statistical analyses. The statistical analysis was performed using STATISTICA software, version 10.0 (StatSoft Corp., Krakow, Poland).

## Results

### Effects of a High-Fat Diet

Administration of a high-fat diet to rats increased the number of NEU in the blood (*P* = 0.014) relative to the group receiving the standard diet (**Table 2**). Feeding rats with a high-fat diet did not affect red blood cell and platelet parameters (**Tables 3**, **4**). While in addition, the high-fat diet caused an increase in the blood levels of IgE (*P* = 0.047), IL-6 (*P* = 0.032), and TNF-α (*P* = 0.022) (**Table 5**).

**Table 2:** Hematological parameters of the blood.

	WBC	LYM	MID	NEU	LYM	MID	NEU
	10^3^/μL	10^3^/μL	10^3^/μL	10^3^/μL	%	%	%
Diet (D)							
S	6.565	5.41	0.395	1.007^b^	78.45	6.165	15.4^b^
F	6.962	5.03	0.405	1.525^a^	72.67	5.927	21.4^a^
Cr source (Cr)							
None	6.885^a^	5.52^a^	0.480^a^	1.37^b^	72.9	7.13^a^	20.0^a^
Cr-Pic	6.905^a^	5.18^b^	0.315^c^	1.41^a^	75.8	4.67^d^	19.5^a^
Cr-Met	6.570^c^	5.05^b^	0.440^a^	1.08^c^	76.9	6.88^b^	16.25^c^
Cr-NPs	6.695^b^	5.13^b^	0.365^b^	1.20^bc^	76.6	5.505^c^	17.9^b^
*P-value*							
D effect	0.113	0.174	0.621	0.014	0.487	0.236	<0.001
Cr effect	0.044	0.017	0.006	0.024	0.084	0.026	0.002
D x Cr interaction	0.053	0.062	0.082	0.126	0.234	0.034	0.046

Data were expressed as mean (for diet—S and F, n = 28; for Cr source—without, Cr-Pic, Cr-Met and Cr-NPs, n = 14).

^a–d^Means differ significantly from the control group at p ≤ 0.05; (to be considered separately for effect of diet (D) and Cr source (Cr); control group for the effect of diet (D)—standard diet (S); control group for the effect of Cr source - diet without of chromium supplementation (None)).

WBC, total white blood cells; LYM, lymphocytes; MID, mid-sized cells; NEU, neutrophils.

S—rats were fed a standard diet; F - rats were fed a high-fat, low-fiber diet; None—rats were fed diets (standard or high-fat, low-fiber) without Cr supplementation; Cr-Pic - rats were fed diets (standard or high-fat, low-fiber) supplemented with 0.3 mg Cr/kg BW from chromium(III) picolinate; Cr-Met—rats were fed diets ((standard or high-fat, low-fiber) supplemented with 0.3 mg Cr/kg BW from chromium(III)-methionine; Cr-NPs—rats were fed diets (standard or high-fat, low-fiber) supplemented with 0.3 mg Cr/kg BW from nano-sized chromium.

**Table 3:** Hematological parameters of the blood.

	RBC	HGB	HCT	MCV	MCH	MCHC	RDWc
	10^6^/μL	g/dL	%	fL	pg	g/dL	%
Diet (D)							
S	8.792	14.07	43.95	50.02	16.0	32.07	18.70
F	8.755	14.20	43.37	49.52	16.2	32.70	19.17
Cr source (Cr)							
None	8.915	14.30	44.25	49.70	16.0	32.20	19.05
Cr-Pic	8.675	14.05	43.25	49.70	16.2	32.55	19.05
Cr-Met	8.830	14.25	43.85	49.75	16.1	32.45	18.85
Cr-NP	8.675	13.95	43.30	49.95	16.1	32.35	18.80
*P-value*							
D effect	0.521	0.338	0.709	0.397	0.458	0.662	0.221
Cr effect	0.125	0.287	0.327	0.782	0.664	0.987	0.072
D x Cr interaction	0.128	0.509	0.367	0.802	0.552	0.391	0.495

Data were expressed as mean (for diet—S and F, n = 28; for Cr source—without, Cr-Pic, Cr-Met and Cr-NPs, n = 14).

RBC, red blood cells; HGB, hemoglobin; HCT, hematocrit; MCV, mean corpuscular volume; MCH, mean corpuscular hemoglobin; MCHC, mean corpuscular hemoglobin concentration; RDWc, red cell distribution width.

S—rats were fed a standard diet; F - rats were fed a high-fat, low-fiber diet; None—rats were fed diets (standard or high-fat, low-fiber) without Cr supplementation; Cr-Pic—rats were fed diets (standard or high-fat, low-fiber) supplemented with 0.3 mg Cr/kg BW from chromium(III) picolinate; Cr-Met—rats were fed diets ((standard or high-fat, low-fiber) supplemented with 0.3 mg Cr/kg BW from chromium(III)-methionine; Cr-NPs—rats were fed diets (standard or high-fat, low-fiber) supplemented with 0.3 mg Cr/kg BW from nano-sized chromium.

### Effects of Different Forms of Cr in the Diet

Compared to the group that did not receive added Cr, the addition of Cr-NPs and Cr-Met to the diet reduced the WBC count (*P* = 0.044), with the lowest value noted for group Cr-Met. Compared to the group that did not receive added Cr, the addition of this element, irrespective of the form used, reduced the LYM count (*P* = 0.017) in the blood, with the lowest value noted in the group receiving Cr-Met. The NEU count was increased by the addition of Cr-Pic to the diet but decreased by the addition of Cr-Met (*P* = 0.024) relative to the group without added Cr. Both Cr-NPs and Cr-Met reduced the NEU percentage (*P* = 0.002) in the blood, with the lowest value noted in the Cr-Met group. The addition of Cr-NPs and Cr-Pic to the diet caused a decrease in the MID count (*P* = 0.006) in the blood of the rats, with the lowest value noted in the Cr-Pic group. In addition, Cr added to the diet, irrespective of the form used, reduced the percentage of MID (*P* = 0.026) relative to the group with no added Cr in the diet, with the most pronounced effect noted in the Cr-Pic group (**Table 2**). Administration Cr-supplemented diet to rats had no effect on red blood cell parameters (**Table 3**).

Both the PLT count (*P* = 0.026) and the platelet percentage PCT (*P* = 0.039) in the blood were decreased by the addition of Cr-NPs, but increased as a result of supplementation with Cr-Met relative to the group without added Cr (None) (**Table 4**).

**Table 4:** Hematological parameters of the blood.

	PLT	PCT	MPV	PDWc
	10^3^/μL	%	fL	%
Diet (D)				
S	625.7	0.512	8.220	35.67
F	623.0	0.507	8.180	35.80
Cr source (Cr)				
None	617.0^b^	0.505^b^	8.180	35.70
Cr-Pic	640.5^ab^	0.525^ab^	8.195	35.80
Cr-Met	650.1^a^	0.530^a^	8.185	35.70
Cr-NPs	589.0^c^	0.480^c^	8.240	35.75
*P-value*				
D effect	0.164	0.309	0.874	0.643
Cr effect	0.026	0.039	0.077	0.654
D x Cr interaction	0.339	0.509	0.261	0.097

Data were expressed as mean (for diet—S and F, n = 28; for Cr source—without, Cr-Pic, Cr-Met and Cr-NPs, n = 14).

^a–c^ Means differ significantly from the control group at p ≤ 0.05; (to be considered separately for effect of diet (D) and Cr source (Cr); control group for the effect of diet (D) - standard diet (S); control group for the effect of Cr source - diet without of chromium supplementation (None)).

PLT, platelet count; PCT, platelet percentage; MPV, mean platelet volume; PDWc, platelet distribution width.

S— rats were fed a standard diet; F - rats were fed a high-fat, low-fiber diet; None—rats were fed diets (standard or high-fat, low-fiber) without Cr supplementation; Cr-Pic - rats were fed diets (standard or high-fat, low-fiber) supplemented with 0.3 mg Cr/kg BW from chromium(III) picolinate; Cr-Met—rats were fed diets ((standard or high-fat, low-fiber) supplemented with 0.3 mg Cr/kg BW from chromium(III)-methionine; Cr-NPs—rats were fed diets (standard or high-fat, low-fiber) supplemented with 0.3 mg Cr/kg BW from nano-sized chromium.

The addition of Cr-Met and Cr-NPs to the diet of rats resulted in an increase in the IgE level (*P* = 0.029) in the blood relative to the group without added Cr. The inclusion of added Cr in the diet, irrespective of its form, increased the blood levels of IgA and IL-2 (*P* < 0.001 and *P* = 0.017, respectively), with the highest values noted in the group receiving Cr-NPs. There was also an increase in the content of IL-6 (*P* < 0.001) in the blood of rats receiving Cr-NPs and Cr-Met, with the lowest level detected following supplementation with Cr-Met. Administration of a Cr-supplemented diet to rats, irrespective of the form used, resulted in an increase in the blood level of TNF-α (*P* < 0.001) relative to the group without added Cr, with the highest value observed in the group receiving Cr-Met. Supplementation with Cr-Pic reduced ceruloplasmin activity (*P* = 0.008) in the blood relative to the group without added Cr, whereas Cr-Met increased the activity of this enzyme. The level of Casp3 in the blood was increased by the addition of Cr-Pic to the diet relative to the group without added Cr (*P* = 0.033), but decreased by Cr-Met. There was also an increase in the Casp8 level (*P* = 0.018) in the blood of rats receiving Cr, irrespective of the form used, with the highest value noted in the group receiving Cr-NPs (**Table 5**).

**Table 5:** Immune parameters of the blood.

	IgE, ng/ml	IgA, ng/ml	IL-2, pg/ml	IL-6, pg/ml	TNF-α, pg/ml	Cp, ng/ml	Casp3, µmol/L	Casp8, µmol/L
Diet (D)								
S	6.754^b^	73.51	13.29	10.55^b^	4.806^b^	2434.7	2.839	5.782
F	7.049^a^	75.92	13.61	17.06^a^	6.878^a^	2451.5	2.984	5.725
Cr source (Cr)								
None	6.602^c^	68.57^c^	12.43^c^	11.81^c^	3.920^d^	2413.5^b^	2.878^b^	5.266^c^
Cr-Pic	6.691^bc^	72.01^b^	13.61^b^	11.44^c^	5.444^c^	2247.1^c^	3.451^a^	5.695^b^
Cr-Met	6.945^b^	73.74^b^	13.31^b^	16.95^a^	7.420^a^	2698.6^a^	2.093^c^	5.662^b^
Cr-NPs	7.368^a^	84.55^a^	14.45^a^	15.02^b^	6.586^b^	2413.1^b^	3.227^ab^	6.392^a^
*P-value*								
D effect	0.047	0.079	0.082	0.032	0.022	0.077	0.067	0.796
Cr effect	0.029	<0.001	0.017	<0.001	<0.001	0.008	0.033	0.018
D x Cr interaction	0.163	0.026	0.051	0.049	0.088	0.039	0.284	0.053

Data were expressed as mean (for diet—S and F, n = 28; for Cr source—without, Cr-Pic, Cr-Met and Cr-NPs, n = 14).

^a–c^Means differ significantly from the control group at p ≤ 0.05; (to be considered separately for effect of diet (D) and Cr source (Cr); control group for the effect of diet (D) - standard diet (S); control group for the effect of Cr source - diet without of chromium supplementation (None)).

IgA, immunoglobulin A; IgE, immunoglobulin E; IL-6, immunoglobulin E; IL-2, interleukin-2; TNF-α, tumour necrosis factor α; Cp, ceruloplasmin; Casp3, caspase 3; Casp8, caspase 8.

S—rats were fed a standard diet; F - rats were fed a high-fat, low-fiber diet; None—rats were fed diets (standard or high-fat, low-fiber) without Cr supplementation; Cr-Pic - rats were fed diets (standard or high-fat, low-fiber) supplemented with 0.3 mg Cr/kg BW from chromium(III) picolinate; Cr-Met—rats were fed diets ((standard or high-fat, low-fiber) supplemented with 0.3 mg Cr/kg BW from chromium(III)-methionine; Cr-NPs—rats were fed diets (standard or high-fat, low-fiber) supplemented with 0.3 mg Cr/kg BW from nano-sized chromium.

## Discussion

### Effects of a High-Fat Diet

Long-term use of a high-fat diet, in both animals and humans, results in the accumulation of fat tissue and an increase in body weight, leading to overweight and obesity (37, 38). Numerous literature data indicate that the development of obesity is accompanied by chronic inflammation in fat tissue (39–41). Influx and accumulation of macrophages from the blood lead to the synthesis and secretion of large amounts of pro-inflammatory substances in fat tissue, predominantly tumor necrosis factor (TNF-α) and interleukin 6 (IL-6). An increased volume of adipocytes increases the secretion of adipocytokines such as leptin and resistin and their concentrations in the blood. These stimulate the secretion of pro-inflammatory cytokines and thereby indirectly exacerbate inflammation (42). In the present study, the blood of rats receiving a high-fat diet contained much higher concentrations of TNF-α and IL-6 than that of rats on a standard diet. There are suggestions that HF diet, by inducing inflammation, may contribute to the increased production of immunoglobulins (43). In our study, an eight-week high fat diet caused a slight increase in the serum concentration of IgA and a significant increase in IgE. Induction of an inflammatory response may also be indicated by an elevated leukocyte count in the blood, which has been observed in a study on mice fed a HF-diet for six months; furthermore, the increase in total leukocyte count was accompanied by an increase in the number of lymphocytes and neutrophils (44). In our experiment, no significant increase in the total WBC count was noted in the blood of rats fed the experimental diet, but an increase in neutrophils count was observed. There were also no changes noted among RBC parameters, in the platelet count, or in parameters characterizing platelets (MPV and PDWc). The study by Maysami et al. (44), cited above, shows that a high proportion of fat in the diet can result not only in an increase in WBC count, but also in a significant increase in parameters such as RBC count, mean corpuscular volume (MCV), red cell distribution width (RDW), Hb level, and Ht value. The lack of such an impact of the diet in our study could result from the fact that the experimental nutrition was used for a relatively short time, i.e. for a period of 8 weeks. An increase in the total number of blood cells (both RBC and WBC) is also observed in the blood of obese individuals, which increases its viscosity, impedes its flow through the blood vessels, and may lead to complications in the form of stroke or other cardiovascular disorders (45). On the other hand, obesity as an effect of a high-fat diet may cause anemia, due to an iron deficiency in the diet and the increased demand for this element resulting from increased blood volume as well as inflammation (46, 47).

### Effects of Different Forms of Cr in the Diet

The evaluation of the effect of different chromium compounds on blood count is necessary to assess their safety for humans and animals, but data in this regard is insufficient. It is known that hexavalent chromium easily penetrates into erythrocytes and may accumulate in these cells, which in turn leads to their damage and various abnormalities (3). Also, trivalent chromium by influencing iron metabolism, may have a negative effect on the process of erythropoiesis and contribute to anemia (1). The scientific literature describes a case of hemolytic anemia and thrombocytopenia as a result of long-term use of high doses of chromium picolinate (far exceeding recommended levels) in weight-loss treatment (48). The results of our study revealed that neither Cr-Pic nor other form of chromium used in the recommended dose caused such effects in HF diet rats (RBC and PLT parameters were unchanged), with the exception of a reduction in the number of thrombocytes in the Cr-NPs group. High biological activity of nanoparticles results from their large chemical reactivity and small size, whereby they easily cross the cell membrane and may affect cell components and their functions (49). Compared to other forms of chromium, Cr-NPs is also better absorbed in the digestive tract (31). It can therefore be presumed that lower doses of chromium in this form are sufficient to induce biological effects, which should be taken into account when assessing the possibility of using NPs as a dietary supplement.

Analyzing the effect of chromium on white blood cell parameters, it was found that the use of Cr-NPs and Cr-Met in HF diet rats substantially decreased the WBC count and reduced lymphocyte and neutrophil cell numbers. Leukopenia may be a consequence of pathologies developing in the bone marrow and causing disturbances of the processes of hemopoiesis. The effects observed in our study could be due to the suppressive effect of Cr on the hemopoietic activity of the bone marrow as determined in study on the toxicity of CrVI in mice (50). It is worth bearing in mind that CrIII may oxidize in the body (especially in the case of ROS overproduction), therefore the effects of both these forms may be similar (51). The decrease in the number of leukocytes in HF diet rats observed in our study may be regarded as a favorable phenomenon, as it may indicate a decrease in the intensity of inflammatory state under the influence of chromium. This is particularly beneficial in conditions accompanied by chronic inflammation and redox imbalance, such as diabetes or obesity (13, 16). Wang et al. (52) in mice with experimentally induced hepatic steatosis showed that chromium decreased TNF‐α, IL‐1β and IL‐12 and augmented IL‐10 level. In studies on streptozotocin-treated diabetic rats (53) and on diabetes patients (17), chromium dinicocysteinate and chromium nicotinate proved most effective as anti-inflammatory agents, while picolinate was least effective in those studies. Jain et al. (54) in a study of monocytes of the U937 line revealed that among three forms of chromium, i.e. chloride, picolinate and niacinate, chromium niacinate most effectively reduced secretion of IL-6 and IL-8, and also reduced the concentration of malonyl dialdehyde. The results of our experiment showed that all chromium supplements contributed to an increase in pro-inflammatory cytokines, nevertheless Cr-Pic was characterized by the weakest activity. Cr-Pic caused an increase in the IL-2 and TNF-α levels but not in IL-6 production. In comparison with picolinate, the other forms of chromium more strongly induced production of pro-inflammatory cytokines, while the main effect of Cr-Met was a significant increase in the levels of IL-6 and TNF‐α. Supplementation with Cr-NPs primarily caused a significant increase in the IL-2 level. It should be noted that IL-2 and TNF-α are cytokines that promote cellular immunity, while IL-6 stimulates the humoral response (55). An increased content of pro-inflammatory cytokines in blood as result of chromium supplementation was observed in another studies (56, 57). The induction of oxidative stress may be considered a probable reason of the pro-inflammatory effects of the tested chromium supplements in rats. Activation of the transcription factor NF-κB that controls the expression of many genes of the inflammatory response, including cytokines (IL-1b, TNFα), chemokines (eotaxin), enzymes (phospholipase A2, lipoxygenase) and some adhesive molecules is mediated by reactive oxygen species (58). The pro-oxidative effect of CrIII, especially in the nanoparticle form, was indicated by our previous study (27) in which it caused intensification of oxidation processes occurring in the brain and heart of HF diet rats. The induction of the inflammatory reaction under the influence of Cr-Met was also evidenced by the increased level of ceruloplasmin, which belongs to the group of acute phase proteins. The increase in the concentration of ceruloplasmin, which has the ability to remove hydroxyl radicals (thanks to the oxidation properties of Fe^2+^ to Fe^3+^), is probably related to the body’s response to the accompanying oxidative stress (59). The main promoter of the synthesis of acute phase proteins is IL-6, the concentration of which was the highest in the blood of rats from Cr-Met group.

Apart from the influence on inflammatory markers, the immunotropic effect of chromium may be manifested by modification of the level of immunoglobulins. Zha et al. (60) demonstrated that in addition to stimulation of the lymphoproliferative response and phagocytic activity of peritoneal macrophages the inclusion of chromium nanoparticles in the diet of rats led to an increase in the plasma IgG. The observed effects the authors explained by the notion that nanoparticles may be recognized by the immune system of animals. The existence of such a possibility is also indicated by Dobrovolskaia et al. (61) and Sembratowicz and Ognik (49). The immunostimulatory effect of chromium is probably linked to improvement in the insulin sensitivity and its inhibitory effect on cortisol synthesis (62). In our study all tested supplements contributed to an increase in the level of immunoglobulins A and E, but the greatest elevation was noted especially in rats receiving chromium in the nano form. An increase in the content of IgE may accompany chronic inflammation or parasitic diseases, but is primarily linked to hypersensitivity reactions (63). Chromium is a known contact allergen, whose symptoms, in the form of skin lesions, appear upon contact with objects containing chromates, i.e. compounds of chromium VI. However, chromium III has also proven capable of inducing an allergic reaction when ingested, with symptoms mainly affecting the skin, in what is known as systemic contact dermatitis. Despite only two such cases have been described (64, 65), the occurrence of hypersensitivity reactions to oral supplementation with CrIII compounds cannot be ruled out.

Another potential adverse biological effect of chromium is pro-apoptotic action. Balamurugan et al. (66), testing the effect of various complexes of chromium on lymphocyte apoptosis, confirmed the important role of ROS in this process. A decreased leukocyte count observed in our study may be associated with induction of their apoptosis by chromium ions. According to Bailey et al. (67), besides causing oxidative stress, the role of chromium in induction of an apoptotic signal may be linked to its effect on DNA. It is known to have potential to react with DNA, cause strand breakage, promote the formation of DNA-protein cross-links, and induce oxidative damage to DNA (68). Proteins known as caspases play a key role in the irreversible effector phase of apoptosis. They can be divided into two groups according to their function: initiator caspases, involved in transmitting apoptotic signals (e.g. caspases 2, 6 and 8), and effector caspases, which directly degrade cellular components *via* proteolytic reactions, leading to their programmed death (caspase 3 and 7) (69). The activity of effector caspases is an indicator of apoptosis in cells and involves regulation mediated by various pro- and anti-apoptotic factors. Petit et al. (70) have shown that the Cr^+3^ ion stimulates expression of caspase 3 and caspase 8 and their activity in human macrophages, which leads to their apoptosis. The authors suggest that induction of this process is the result of the interaction of Cr^+3^ with cell membrane components, which in turn contributes to activation of initiator caspase 8. Also responsible for the pro-apoptotic effect of chromium against macrophages, at least in part, is its capacity to inhibit expression of Bcl-2 (an anti-apoptotic protein) and to induce expression of Bax (a pro-apoptotic protein). In our study, all forms of chromium increased the content of caspase 8 in the blood of the rats, with the greatest increase caused by chromium nanoparticles. In the case of caspase 3, an effector caspase, its increase in the blood was most strongly induced by Cr-Pic. The special role of chromium picolinate in this respect may be due to the fact that it contains an aromatic two-donor ligand, so that chromium in this form is more susceptible to reducing agents than in other compounds and can enter into the Fenton or Haber-Weiss reaction (33). Cr-Pic penetrates inside the cell as a complex, which may facilitate its further reduction and increasing generation of reactive oxygen species (67). As no studies have been conducted to test the effect of nano-chromium on apoptosis, we can only rely on the results of experiments on other metal nanoparticles. According to Ma and Yang (71), they can trigger apoptosis through a variety of mechanisms, including ROS-induced oxidative stress, internal pathways associated with mitochondrial stress and endoplasmic reticulum stress, and also an external pathway associated with death receptor activation.

## Conclusions

Feeding rats a high-fat diet led to an increase in markers of induction of inflammation, i.e. pro-inflammatory cytokines IL-6 and TNF-α, as well as a significant increase in IgE. The diet did not influence blood counts, except for an increase in the number of granulocytes and their percentage share of the leukogram. The chromium compounds analyzed, especially Cr-Met and Cr-NP, stimulated the immune system of the rats, as indicated by the increased concentrations of IgA, IgE, IL-2, IL-6, TNF-α, and Cp. Given the increase in inflammatory mediators induced by chromium, it should not be used to mitigate the effects of a high-fat diet. Moreover, chromium picolinate and chromium nanoparticles were shown to increase the content of caspase 3 and 8 in the blood of rats, which indicates a pro-apoptotic effect. The effects of the use of chromium nanoparticles include reductions in the WBC count and in the thrombocyte count (leuko- and thrombopenia). Taking account these data the use of chromium as dietary supplement should be reconsidered.

## Data Availability Statement

The datasets presented in this study can be found in online repositories. The names of the repository/repositories and accession number(s) can be found in the article/supplementary material.

## Ethics Statement

The animal study was reviewed and approved by Local Animal Care and Use Committee (Approval No. 04/2019; Olsztyn, Poland).

## Author Contributions

WD, JJ, and KO conceived and designed the experiments. JJ and BF performed the experiments. WD, IS, EC, and KT analyzed the data. WD, IS, EC, BF, and KT contributed reagents/materials/analysis tools. WD, IS, EC and KT wrote the manuscript. JJ and KO reviewed the manuscript. All authors contributed to the article and approved the submitted version.

## Conflict of Interest

The authors declare that the research was conducted in the absence of any commercial or financial relationships that could be construed as a potential conflict of interest.
